# Alcohol and cannabis use as risk factors for injury – a case-crossover analysis in a Swiss hospital emergency department

**DOI:** 10.1186/1471-2458-9-40

**Published:** 2009-01-29

**Authors:** Gerhard Gmel, Hervé Kuendig, Jürgen Rehm, Nicolas Schreyer, Jean-Bernard Daeppen

**Affiliations:** 1 Alcohol Treatment Center, Lausanne University Hospital, Lausanne, Switzerland; 2 Swiss Institute for the Prevention of Alcohol and Drug Problems, Lausanne, Switzerland; 3 Research Institute for Public Health and Addictions, Zurich, Switzerland; 4 Centre for Addiction and Mental Health, Toronto, Ontario, Canada; 5 Public Health Sciences, Faculty of Medicine, University of Toronto, Canada; 6 Clinical Psychology and Psychotherapy, Technische Universität Dresden, Dresden, Germany; 7 Interdisciplinary Center of Emergencies, Lausanne University Hospital, Lausanne, Switzerland

## Abstract

**Background:**

There is sufficient and consistent evidence that alcohol use is a causal risk factor for injury. For cannabis use, however, there is conflicting evidence; a detrimental dose-response effect of cannabis use on psychomotor and other relevant skills has been found in experimental laboratory studies, while a protective effect of cannabis use has also been found in epidemiological studies.

**Methods:**

Implementation of a case-crossover design study, with a representative sample of injured patients (N = 486; 332 men; 154 women) from the Emergency Department (ED) of the Lausanne University Hospital, which received treatment for different categories of injuries of varying aetiology.

**Results:**

Alcohol use in the six hours prior to injury was associated with a relative risk of 3.00 (C.I.: 1.78, 5.04) compared with no alcohol use, a dose-response relationship also was found. Cannabis use was inversely related to risk of injury (RR: 0.33; C.I.: 0.12, 0.92), also in a dose-response like manner. However, the sample size for people who had used cannabis was small. Simultaneous use of alcohol and cannabis did not show significantly elevated risk.

**Conclusion:**

The most surprising result of our study was the inverse relationship between cannabis use and injury. Possible explanations and underlying mechanisms, such as use in safer environments or more compensatory behavior among cannabis users, were discussed.

## Background

There is sufficient evidence indicating alcohol use as a contributing causal factor for injury [1,2]. This evidence comes from case-control (e.g., [3,4]), case-crossover (e.g., [4-6]), and experimental studies [7,8]. While most of the research has been conducted on traffic injuries, there is also ample evidence for the role of alcohol in other mechanisms of injuries, including clear biological pathways via effects on the central nervous system and resulting behavioural consequences, even at relatively low levels of consumption [8].

Meanwhile, the causal relationship between cannabis use and injury is less clear. While there is consistent evidence from laboratory studies of a dose-response effect of cannabis use on psychomotor and other relevant skills [9-13], the epidemiological evidence is still inconsistent [14]. Most of this research has been conducted in the area of driving impairment from the influence of cannabis (but see [15]). In a major review of 17 studies from around the world, Macdonald and colleagues [16] reported that the average proportion that tested positive for cannabis use across studies was 7.8% for fatally injured drivers (ranging from 1.4% to 27.5%) and 11.9% for non-fatally injured drivers (ranging from 5% to 16.9%). However, as cannabis screening tests may show positive results for days or weeks after a single usage [17,18], clearly long after the drug's period of influence over psychomotor behaviour has ended [18], such data are of limited value when attempting to assess the actual causal impact of cannabis use on collision risk. Moreover, as control conditions were often not included in theses studies, the mere presence of cannabis in a certain proportion of people injured in traffic collisions did not establish causality. In addition there is a lack of consensus among studies that have employed control groups or conditions, as some suggest a causal relationship between cannabis consumption and injury, while others do not [19-21].

Furthermore, in cases where cannabis was established as a risk factor for fatal injury, there were problems with the control group, such as in the study of Dussault and colleagues [20] where only 49.6% of controls agreed to provide consent for a urine sample for analysis. Substance use and injury studies generally have the difficulty defining an adequate control group (e.g., non-injured as controls for injured [22]). One way of overcoming this problem is responsibility analysis, which is based on the premise that if cannabis use increases collision risk, than the drug should be more likely to be detected in drivers judged responsible for collisions, as compared to other drivers [23]. However, the overall results of responsibility analysis have been mixed [10,24-26].

In summary, the aforementioned laboratory studies have indicated the causal influence of cannabis in psychomotor skills impairment. While it is not clear to what degree this effect is relevant in real-life situations, it has been proposed that people driving under the influence of cannabis may actually recognize the impairment and compensate, thus becoming more cautious in real-life driving situations [10,24]. Nevertheless, as is the case for alcohol, there should be limits on such compensation [27,28].

The combination of alcohol and cannabis use for injury is potentially an important public health question for two reasons. First, given the substantially increase in prevalence of cannabis use in Switzerland, as in many other countries over the past years, the prevalence of combined use may have also increased likewise. Second, combined alcohol and cannabis use may result in multiplicative, rather than additive, risks. Some studies in the area of traffic injuries have indicated such a multiplicative effect (e.g., [21]), but as with other research in this field, findings have been inconsistent.

The main objective of this research is to assess the potential impact of alcohol and cannabis use on risk for injury. Specifically, we hypothesize that:

• The higher the alcohol use, the higher the subsequent risk of injury.

• Cannabis use will increase the subsequent risk of injury.

• Combined use of alcohol and cannabis will result in a higher risk of injury than expected in a purely additive model.

The present study was planned to expand on existing research in several ways:

• We did not restrict ourselves to traffic injury, but included all categories of injury.

• We used case-crossover design to avoid problems in comparability of cases and controls. This methodology uses each injured person as his/her own control, by assessing the relevant exposure immediately before the event, as well as of the same period one week prior.

## Methods

### Sample and design

This study used a case-crossover design, with respondents serving as their own controls [29,30]. A representative sample of injured patients (N = 486; 332 men; 154 women) treated in the Emergency Department (ED) of the Lausanne University Hospital (CHUV; Switzerland) was collected. The CHUV is the main public hospital in the Lausanne area (pop. 200,000) and is thus expected to process the majority of injured patients. Alternative trauma care consisted of private medical practices and medical day care centers, both of which were used for medical rather than trauma reasons due to insufficient infrastructure. Given this situation, all severe and common injuries that occur in the Lausanne area expect to be diagnosed and possibly treated at the ED of the CHUV. For the collection of data, 270 out of more than 900 four-hour time slots were randomly chosen during five one-month periods, between September 2005 and July 2006. All hours (24/24) of all days (7/7) of the week were included in the sampling. Criteria for inclusion were: a) being aged sixteen or older, b) sufficient understanding of the French language, c) attendance of the ED within twenty-four hours of the occurrence of injury, and d) no attendance of the ED for follow-up care. According to administrative statistics, 1,165 injured patients were admitted to the ED during the field phase. Among them, 391 (33.6%) were not eligible according to the inclusion criteria, i.e. age or language (n = 87), late attendance or follow-up care (n = 304). Other reasons for non-inclusion included vetoes from the medical staff (i.e. patients very severely injured or accompanied by police or security staff, n = 26); death after admission (n = 4); no possibility for informed consent (i.e. patients with a degenerative brain disorder, n = 37); unrecorded reason (n = 1). Of the remaining 706 eligible patients, 43 could not be interviewed due to a rapid transfer to other wards, out of the ED, where it was impossible to conduct interviews. In addition, 115 could not be interviewed due to interviewer work overload or ongoing medical care. Three patients refused any collaboration and 57 actively refused to participate in the study (8.1% of total eligible sample). Thus, non-response (response rate = 69.1%) was due mostly to organizational issues and refusal was rare (the cooperation rate excluding those not contacted due to interviewer work overload or ongoing medical care was 82.6%). Finally, two patients had incomplete data on either alcohol or cannabis use and were thus excluded from the analysis. As a result the final sample included N = 486 patients. The study was approved by the Ethics Committee for Clinical Research of the Lausanne University Medical School (No. 96/05).

### Measures

The case-crossover design used the six-hour period preceding the injury event as the case period and the same six-hour period of exactly one week prior to the event as the control period. The questionnaire followed the protocol developed by the WHO Collaborative Study Group on Alcohol and Injuries [31], with adaptations for questions on cannabis use. Alcohol consumption within the six-hour periods was measured in drinks of different container sizes (e.g., small bottle of beer = 0.3 l or a can of beer = 0.5 l) for beer, wine, champagne, aperitifs, spirits, alcopops (lemonade drinks premixed with spirits), and mixed drinks such as cocktails. The number of drinks along with their respective container sizes were converted into grams of pure ethanol using the official conversion rates of the Swiss Alcohol Board (11 vol. % for wine, 4.8 vol. % for beer and 40 vol.% for spirits) and summed across beverages. Alcohol use was then broken down into four categories: 1) no use; 2) less than 20 grams for men, respectively less than 10 grams for women; 3) 20 to 40 grams and 10 to 30 grams (men/women); and 4) more than 40 grams, respectively more than 30 grams (men/women). Cannabis use within the six-hour periods (case and control) was measured through a single quantity question and similarly broken down into four categories: 1) no use; 2) "less than a pipe or joint"; 3) "about a pipe or joint"; and 4) "more than a pipe or joint".

In addition, self-reports were corroborated with alcohol exposure measured in blood samples from a consenting sub-sample. Only those individuals (n = 356) who arrived within the six-hour period of injury were approached, of which 126 gave permission and were tested. Two sets of samples for blood plasma and serum were taken and stored immediately in a refrigerator in the ED, rapidly frozen later that same day in the University Institute of Forensic Medicine, and analysed within the month. Levels of ethanol in the blood or breath and markers of ethanol use in the serum were tested (results on markers were not used in this current study).

Cannabis use was detected through testing for three blood components: tetrahydrocannabinol (THC), and its metabolites 11-hydroxy-tetrahydrocannabinol (11-OH-THC) and carboxy-tetrahydrocannabinol (THCCOOH). A person tested positive for cannabis use if one of the three measures exceeded the limit of detection of 0.5 ng/ml. It should be noted that we were well aware that positive results for the metabolite THCCOOH only indicates usage at some point in the previous few weeks (12), and is not an indicator of possible impairment at the time of the injury [32]. Risk relationships were based on self reported cannabis use.

### Statistical analysis

A matched-pair case-crossover design was used for the statistical analysis [30]. The estimate of a relative risk with dichotomous outcomes is the ratio of discordant pairs of a case (period) by control (period) in a 2 by 2 table, i.e. a division of the number of users in the six-hour case period (that were not so in the control group) by the number of users in the week prior (control period) that were not users in the six hours prior to injury (case period). This estimator is equivalent to the estimator obtained in a conditional logistic regression where the strata are the individuals [33]. Conditional logistic regression was then used to obtain confidence intervals, this also permitted the estimation of more complex dose-response (levels of substance use) and interaction models (joint effect of alcohol and cannabis use).

## Results

The distribution of injuries in our sample of 486 patients included 45% fall injuries, 15% motor vehicle injuries, 9% intentional injuries, and 30% other injuries (1% missing values). More patients reported alcohol use in the six-hour period prior to injury (case period) than in the corresponding six-hour period in the previous week (control period). Consumption levels among users were on average, higher for the case period than for the control period (Table 1). For cannabis, fewer people reported use prior to injury (case period) than in the control period, with the level of use among users, on average, lower in the case period. The same was found for cannabis use in combination with alcohol use. It should be noted that only 14 men and no women of our sample had used cannabis before the injury.

**Table 1:** Alcohol- and cannabis use characteristics 6 hours prior to injury and in the control period

		**men (n = 332)**	**women (n = 154)**	**total (n = 486)**
**Drinking**				

Just prior to injury	yes	28.3	16.9	24.7

A week prior to injury	yes	19.6	11.0	16.9

**Drinking levels among user**				

Just prior to injury	low	30.9	30.8	30.8

	medium	21.3	26.9	22.5

	high	47.9	42.3	46.7

A week prior to injury	low	51.7	50.0	51.3

	medium	20.0	31.3	22.4

	high	28.3	18.8	26.3

**Cannabis use**				

Just prior to injury	yes	4.2	0.0	2.9

A week prior to injury	yes	6.3	1.9	4.9

**Use levels among user**				

Just prior to injury	< 1 joint/pipe	42.9	n.a.	29.3

	1 joint/pipe	35.7	n.a.	24.4

	> 1 joint/pipe	21.4	n.a.	14.6

A week prior to injury	< 1 joint/pipe	14.3	0.0	12.5

	1 joint/pipe	42.9	33.3	41.7

	> 1 joint/pipe	42.9	66.7	45.8

**Joint use**				

Just prior to injury	none	69.0	83.1	73.5

	alcohol only	26.8	16.9	23.7

	cannabis only	2.7	n.a.	1.9

	both	1.5	n.a.	1.0

A week prior to injury	none	76.5	87.7	80.0

	alcohol only	17.2	10.4	15.0

	cannabis only	3.9	1.3	3.1

	both	2.4	0.6	1.9

Alcohol consumption:

low: less than 20 grams for men and 10 grams for women;

medium: 20 to 40 grams for men, 10 to 30 grams for women;

high: more than 40 grams for men, more than 30 grams for women;

n.a. not applicable; no cannabis use 6 hours prior to injury among women.

As can be seen in Table 2, any alcohol use prior to injury was associated with an increased risk for injury (but did not reach the 5% significance level for women). Relative risks increased with a dose response relationship with increasing alcohol use in both sexes. However, only high alcohol use in women, and medium and high alcohol use in men were significantly different from no alcohol use.

**Table 2:** Relative risk estimates for any alcohol use and its dose-response relationship

	**Women**			**Men**			**Total**		
	**RR**	**95% CI lower**	**95% CI upper**	**RR**	**95% CI lower**	**95% CI upper**	**RR**	**95% CI lower**	**95% CI upper**

**drinking status 6 hours before injury status**									

No (reference)	1			1			1		

Yes	2.50	0.97	6.44	3.23	1.73	6.02	3.00	1.78	5.04

**dose response (women/men)**									

no alcohol use (reference)	1			1			1		

Low	1.00	0.29	3.45	1.49	0.66	3.36	1.37	0.69	2.70

medium	6.41	0.76	54.20	2.91	1.16	7.32	3.14	1.41	6.97

High	25.26	1.66	383.62	7.80	2.86	21.26	8.97	3.55	22.69

Alcohol consumption:

low: less than 20 grams for men and 10 grams for women;

medium: 20 to 40 grams for men, 10 to 30 grams for women;

high: more than 40 grams for men, more than 30 grams for women.

Conversely, cannabis use was associated with a significantly lowered risk for injury (Table 3). Whereas the risk for injuries associated with the use of less than a pipe or joint's worth were not significantly different from the one associated with no use, relative risks decreased with increasing levels of use and were significantly lower than 1.

**Table 3:** Relative risk estimates of any cannabis use prior to injury and dose response estimates

	**RR**	**95% CI lower**	**95% CI upper**
**cannabis use**			

no	1		

yes	0.33	0.12	0.92

**dose response**			

no use	1		

less than 1 pipe or joint	1.45	0.34	6.23

1 pipe or joint	0.11	0.01	0.89

more than 1 pipe or joint	0.03	0.00	0.44

There were no women using cannabis in the 6 hours prior to injury and only 3 women a week ago. Therefore, only results for the total sample were reported

As shown in Table 4, alcohol was the major risk factor for injury and remained significant even after adjusting for cannabis use and the interaction between alcohol and cannabis use. The prevalence of users of both alcohol and cannabis was low (i.e. 1.0% in case period and 1.9% for the control period, see table 1). The findings in Table 4 indicate that cannabis use in combination with alcohol use did not increase risk for injury.

**Table 4:** Relative Risk estimates for alcohol and cannabis use 6 hours prior to injury

	**RR**	**95% CI lower**	**95% CI upper**
no use	1.00		

alcohol use only	3.08	1.77	5.34

cannabis use only	0.37	0.10	1.43

joint use of alcohol and cannabis	0.71	0.12	4.26

There were no women using cannabis in the 6 hours prior to injury and only 3 women a week ago. Therefore, only results for the total sample were reported

We also asked about the use of the following substances, other than alcohol and cannabis, in the period prior to the injury: cocaine, benzodiazepines or drugs with similar properties, the latter was based on a list of the 25 most common of these medicines in Switzerland; as well as added an open-ended question on (other) drugs, which resulted in mentions of methadone, ecstasy, and other psychotropic pharmaceuticals. 9% of the sample indicated use of such drugs. The proportions were 12% in the group with alcohol use without cannabis use, 0% in the group with cannabis use without alcohol use, and 20% in the group with combined use of alcohol and cannabis.

Regarding the validity of self-reporting alcohol use, patients were ten times more likely to have self-reported alcohol use while having negative blood alcohol concentrations, compared with having a positive blood alcohol concentration while self-reporting no alcohol use (for details see [34]). With cannabis use, 113 individual had negative matches (no self reported use, no positive blood test). Three individuals (of 14) with self-reported use in the case period provided blood samples, 2 of which were positive. Of the remaining 10 individual's blood samples, 8 were positive; of which 3 reported use in the control period, leaving 5 with a positive blood screen and no self-reported use in either the case or control period. There were also 2 who reported cannabis use in the control period but were negative on their blood samples. These data did not give strong support for the hypothesis of deliberate denial. It should be noted that the 5 individuals, even with positive screens and no use in either the case or the control period, were were not necessarily persons who denied use when they had in fact used. Cannabis use in the blood can be detected from usage prior to the reference period used here, and of course, there may have been usage in between.

## Discussion

The results of our study corroborate research showing the detrimental effect of alcohol use on injury. The results for cannabis use were quite surprising, as they were associated with less risk of injury, which seems to contradict the laboratory studies cited above, as well as opposes the observations on alcohol use. While these findings are thus more in line with other epidemiological studies showing no effect of cannabis use, possibly even a protective one (e.g., [32,35]), they are limited by the small sample size of cannabis users and do not necessarily contradict findings from laboratory studies. One possibility is that persons driving under the influence of cannabis become more cautious in real-life driving situations than they would in the laboratory [10,24], e.g., by avoiding potentially risky situations [10]. Furthermore, the present study did not only look at traffic casualties, but also included other mechanisms for injury with potential links to cannabis use that are different from those for traffic injuries.

The compensation hypothesis is unlikely to be the only explanation of our results. First, as is the case for alcohol, there should be limits for such compensation [27,28]. The present study in fact indicated a 'protective effect' of cannabis use in a dose-response relationship. Second, the combined intake of alcohol and cannabis failed to show an increased risk for injury when compared with unaccompanied alcohol use, as should be expected [21]. Nonetheless, the relative risk was below even 1 when compared with individuals that abstained from both substances in the six-hour period prior to injury. Third, compared with other studies (e.g., [16,21]) only very few (i.e. 2.9% of the present sample) had consumed cannabis in the six-hour period prior to injury. This seems to be quite low given the fact that Switzerland has one of the highest cannabis use prevalence rates in the world (see [36] for students, and [37] for adults), and therefore may point to the possibility that Swiss cannabis users take precautions to avoid injuries while using cannabis.

Another possible explanation is that when compared to alcohol consumption, cannabis is consumed in relatively safer, low risk environments (e.g. at home, private locations, not public such as bars or while 'going out'), independent of whether also it is consumed with alcohol. Future studies should look at the environment of predominant cannabis usage and whether the place of consumption (e.g. at home, during special occasions) is differentially associated with risks of injuries. The number of cannabis usage cases in the present study was simply too small, to permit a stratified analysis by place of consumption.

As the main results of the study are based on self-reported measures of cannabis use, there may have been a deliberate denial of illicit behaviour. Although this may explain the small prevalence, it is unlikely to explain a protective association with injury. It also begs the question of why individuals would deny cannabis use before an injury, but admit use in the week prior (which is needed to result in a relative risk below 1)? As shown above, there is not a lot of indication for deliberate denial. However, there may be other memory effects, e.g., the ability for recall may have been impeded by alcohol and cannabis use, especially for the period of one week before the injury. Recall errors may have also particularly affected the estimates in case-crossover studies (e.g., overestimation of relative risks due to the underestimation of use in the control period that is retrospectively farther away in time [38]), but would not explain why cannabis use had a protective effect.

While the participation rate was relatively high (8% refusals), we cannot exclude the possibility, that exclusion may have been associated with exposure. Another limitation of our research was the small sample size for cannabis use, and as a result, we should be cautious with conclusions. Clearly, more research is needed, epidemiological studies in particular. Roadside surveys in the tradition of Borkenstein and colleagues [3] should be conducted more systematically to allow for a better examination of the causal effects of cannabis use in traffic injury. In such studies, not only is the blood alcohol concentration in participants of traffic injuries measured, but a random sample of comparable traffic participants is measured as well. This allows for the establishment of relative risk estimates. However, even if such studies yield significant relative risks for injury as a consequence of cannabis use, this does not necessarily demonstrate sufficient evidence for the public health importance of the problem. It may be that although cannabis is causally connected to traffic injury, the importance of this risk factor is small due to a low prevalence of people combining cannabis with other risk factors, such as drinking and driving. Thus, attributable fractions may be small. Of course, the public health importance may differ in regions with higher prevalence of cannabis use. One of the more interesting research continuations in this area would be to replicate this study in regions with higher prevalence of cannabis use and higher incidence of injury (e.g., some parts of Africa or the Caribbean).

In conclusion, we need a more systematic exploration of the relationships between cannabis use and other forms of injury than traffic injuries. There are some indications of cannabis use having a negative biological link to aggression [39], hence many categories of injuries may be differentially impacted by cannabis as compared to alcohol. These relationships should be studied in real life settings and not only in the laboratory, as other factors such as deviance or stigmatization may play a role.

Public health researchers tend to disregard subjective reports of cannabis users, seemingly indicating that they are more cautious and avoid risky situations subsequent to use (e.g., [40]). However, given the numerous reports on different mechanisms of injury, we should take these reports more seriously and should start exploring more systematically which situations involving cannabis use are associated with increased or decreased risks of injury. Such research could not only shed light on important interactions between social and biological determinants of behaviour, but could also contribute to better preventing cannabis-related harm.

## Conclusion

This study indicated a 'protective' effect of cannabis use on injury incidence, in a dose-response relationship, whereas alcohol use showed a detrimental effect. Cannabis use in combination with alcohol, did not increase risk compared to alcohol use alone. Clearly, the findings are not sufficient for calling change in legal limits for cannabis use e.g. in road traffic laws, as laboratory studies have conversely shown that cannabis use causes impairment of psychomotor skills. The findings do call, however, for a more attentive analysis of the environment in which cannabis use takes place and its behavioral consequences, both of which may be associated with avoiding situations with an increased risk of injuries.

## Competing interests

The authors declare that they have no competing interests.

## Authors' contributions

GG drafted the first version of the manuscript, developed the statistical analysis plan and participated in statistical analysis. HK organized data collection and fieldwork of interviewers, performed data preparation and editing, performed statistical analysis, and revised the manuscript for important intellectual content. JR developed conception and design of the study, contributed most parts of the interpretation of findings, and revised the manuscript for important intellectual content. NS was responsible for data acquisition in the ED, the implementation and adaptation of the design in the ED, and contributed to the interpretation of findings. JBD was responsible for all organizational aspects of the coordination between research group and medical staff in the ED, participated in statistical analysis and in drafting and revising the manuscript. All authors have given final approval of the version to be published.

## Pre-publication history

The pre-publication history for this paper can be accessed here:


